# Unravelling the bioherbicide potential of *Eucalyptus globulus* Labill: Biochemistry and effects of its aqueous extract

**DOI:** 10.1371/journal.pone.0192872

**Published:** 2018-02-13

**Authors:** Carolina G. Puig, Manuel J. Reigosa, Patrícia Valentão, Paula B. Andrade, Nuria Pedrol

**Affiliations:** 1 Department of Plant Biology and Soil Science, University of Vigo, Vigo, Spain; 2 REQUIMTE/LAQV, Laboratory of Pharmacognosy, Department of Chemistry, University of Porto, Porto, Portugal; Universita degli Studi di Napoli Federico II, ITALY

## Abstract

In the worldwide search for new strategies in sustainable weed management, the use of plant species able to produce and release phytotoxic compounds into the environment could be an effective alternative to synthetic herbicides. *Eucalyptus globulus* Labill. is known to be a source of biologically active compounds responsible for its phytotoxic and allelopathic properties. Our previous results demonstrated the bioherbicide potential of eucalyptus leaves incorporated into the soil as a green manure, probably through the release of phytotoxins into the soil solution. Thus, the aims of this study were to understand the phytotoxicity of the eucalyptus leaves aqueous extract applied in pre- and post-emergence, and to identify and quantify its potentially phytotoxic water-soluble compounds. The effects were tested on the germination and early growth of the model target species *Lactuca sativa* and *Agrostis stolonifera*, and on physiological parameters of *L*. *sativa* adult plants after watering or spraying application. Dose-response curves and ED_50_ and ED_80_ values for eucalyptus aqueous extracts revealed pre-emergence inhibitory effects on both target species, effects being comparable to the herbicide metolachlor. While spraying treatment reduced the aerial and root biomass and increased the dry weight/fresh weight ratio of lettuce adult plants, watering application reduced protein contents and chlorophyll concentrations with respect to control, reflecting different modes of action depending on the site of phytotoxin entry. Via HPLC analyses, a total of 8 phenolic compounds (chlorogenic, two ρ-coumaric derivatives, ellagic, hyperoside, rutin, quercitrin, and kaempferol 3-O-glucoside) and other 5 low weight organic acids (citric, malic, shikimic, succinic and fumaric acids) were obtained from aqueous extract, the latter being identified for the first time in *E*. *globulus*. Despite some phytotoxic effects were found on lettuce adult plants, the use of eucalyptus aqueous extract would be discarded in post-emergence, whereas it was promising as a pre-emergence bioherbicide.

## Introduction

During the past years, an increasing demand for the environmental safety of pesticides has led to looking for new nonsynthetic management methods in agriculture. In this context, sustainable weed management is one of the main challenges for both organic and conventional agriculture [1]. However, the existing products on the market targeting weeds are weakly developed compared with those targeting other pests and diseases [1]. Hence, in the necessary search for new strategies for weed control in organic agriculture, the use of plant species able to produce and release phytotoxic compounds could be an effective tool.

The first step to find possible candidates as bioherbicides consists in assessing the phytotoxic activity of plant material aqueous extracts on morpho-physiological parameters of model target species, by *in vitro* bioassays. Phenolic compounds, together with other low molecular weight organic acids, are present in plant aqueous extracts because of their water-soluble nature, and several studies have demonstrated their phytotoxic potential against different target species [2,3]. The differential allelopathic effect of each plant species would depend on the type and amount of individual phytotoxic compound as well as on the mixture of compounds present in the plant extract [4,5]. Therefore, chemical identification procedures such as chromatographic techniques for analysis of phenolic compounds have been thoroughly improved in the last years [6].

Evidence of the allelopathic activity and chemical composition of different species of the genus *Eucalyptus* (Myrtaceae) have been widely reported. Plants of the genus *Eucalyptus* possess strong pesticide potential due to the presence of a wide array of biologically active compounds, especially from their essential oils rich in terpenoids [7,8]. The phytotoxic effects of *Eucalyptus globulus* Labill. aqueous extracts or leachates have been demonstrated on the germination and radicle growth of some crops such as sorghum (*Sorghum vulgare*) [9], *Brassica* sp. [10], eggplant (*Solanum melongena*) [11], barley (*Hordeum vulgare*) [12] or fingermillet (*Eleusine coracana*) [13], as well as on weed species: purple nutsedge (*Cyperus rotundus*) and bermudagrass (*Cynodon dactylon*) [14], or redroot pigweed (*Amaranthus retroflexus*) and barnyardgrass (*Echinochloa crus-galli*) [15]. Our previous results in greenhouse conditions demonstrated the bioherbicide potential of eucalyptus leaves incorporated into the soil as a green manure, pointing out different modes of action for target species and attributing the phytotoxic effects probably to the release of water-soluble compounds into the soil solution [15]. Therefore, it is time to detail the chemical composition of *E*. *globulus* aqueous extract and to determine the primary physiological processes affected in target species.

Several studies on the phenolic composition of *E*. *globulus* leaf aqueous extracts revealed the presence of different hydroxycinnamic and hydroxybenzoic acids: caffeic, ferulic, and chlorogenic, as well as some flavonoids: rutin, quercitin, hyperoside and ellagic acid, but these studies were usually related to an antioxidant activity [6,16–19]. However, only some isolated reports deal with the relation between the phenolic composition of the leaf extracts and their allelopathic properties [20–22]. To our knowledge, no reports have been done to study the organic acid composition from *E*. *globulus* leaf aqueous extract. Furthermore, the important stock of eucalyptus plantations, mainly devoted to paper industry, present in the agroecosystems (and the fact that it is one of the most world widespread species) makes *E*. *globulus* biomass an optimal candidate for its potential use as a bioherbicide.

For this work, *E*. *globulus* aqueous extract was evaluated for its allelopathic potential as natural herbicide, by the following objectives: i) to assess the phytotoxicity of aqueous extract applied in pre- and post-emergence, and to determine the primary physiological processes affected; and ii) to identify potentially phytotoxic compounds present in the aqueous extract.

## Materials and methods

### Plant material and aqueous extracts

Fresh eucalyptus adult leaves from *Eucalyptus globulus* Labill. were collected from several 10 years old plantations surrounding University of Vigo (Galicia, NW Spain, 42.09°N, 8.25°W), and slashed in 1 cm^2^-sized pieces immediately after collection. Extractions were performed in 2-L Erlenmeyer flasks at a plant dry weight/distilled water volume ratio of 66.7 g L^-1^ (the maximum ratio to keep a volume of chopped plant material completely soaked). Dry weight/fresh mass ratio was obtained by drying aliquots of fresh material at 70°C for 72 h. Flasks were maintained in the dark at room temperature for 24 h, being gently soaked every 6 h. The aqueous extracts were filtered through cellulose membrane 0.45 μm pore size to clear impurities and then through 0.2 μm to sterilize the extract. The extracts were then frozen at -20°C in sterile plastic bottles until bioassayed.

For the bioassays, lettuce (*Lactuca sativa* L. cv. `Great Lakes´) and bentgrass (*Agrostis stolonifera* L. cv. Penncross) were chosen respectively as dicot- and monocotyledon ‘standard target species’ (STS), thoroughly used in literature for their fast germination, genetic homogeneity, and high sensitivity to allelochemicals [23,24], thus allowing comparisons for many different compounds [25]. Seeds of lettuce and bentgrass were purchased from Fitó S. A. (Barcelona, Spain) and Rocalba S. A. (Girona, Spain), respectively.

### Pre-emergence dose-response bioassays

The crude aqueous extract was successively diluted in distilled water to get five final concentrations, those corresponding to 66.7, 33.3, 16.7, 6.7 and 0 g L^-1^ on a dry mass basis. Values for pH (CrisonMicropH 2001 pH-meter), electrical conductivity (EC, Crison CDTM-523 conductivity meter), and osmolarity (Gonotec OSMOMAT 030 cryoscopic osmometer) corresponding to each dilution were: 4.75, 4.44, 4.62, 4.87 and 6.55; 1.37, 0.70, 0.40, 0.16 and 0.00 dS m^-1^; and 0.064, 0.037, 0.016, 0.006 and 0.000 osmol kg^-1^, respectively. The synthetic pre-emergence herbicide S-metolachlor 960 g L^-1^ (Dual Gold^®^, Syngenta Agro S.A., Madrid, Spain) was prepared at 0.153 g L^-1^ (540 μM) in such a way attaining the label dose (LD, 0.96 kg ha^-1^), and used as positive control. Values of pH, EC and osmolarity were 6.14, 0.02 dS m^-1^ and 0.099 osmol kg^-1^. Lettuce seeds were incubated in 6-well dishes, at a rate of 15 seeds per well for germination bioassay, and placed on a filter paper wetted with 600 μL of solution. Dishes were sealed with Parafilm and incubated in the dark at 27°C and a relative humidity of 75% during 48 hours. The number of germinated seeds (rupture of seed coats and the emergence of radicle ≥ 1 mm [26]) was counted every 24 hours until no further seeds germinated. Total percentage of germinated seeds (Gt) was calculated from the cumulative germination data [27]. For radicle growth bioassay, lettuce seeds were pre-germinated on wet filter paper in plastic trays filled with perlite watered at saturation with can water and incubated at 27°C for 24 h. After incubation, ten pre-germinated seeds (radicle length between 1–3 mm [26]) were transferred to each well dish as described above and placed in a growth chamber at 25/20°C, 18/6 h light/dark. After 48 h of incubation, radicle length was measured. Each treatment was replicated five times.

Phytotoxicity bioassays with bentgrass were carried out according to the procedure described by Dayan *et al*. [28]. Twelve mg of *A*. *stolonifera* seeds were placed on a Whatman 3 MM Chr filter paper in each well of a 24-well plate. Two hundred fifty μL of the different aqueous extract concentrations and metolachlor were added at random to different wells. Plates were incubated at 27°C in continuous light. Phytotoxicity was evaluated by visually comparing the germination and seedling growth in each well after 12 days. The phytotoxicity was rated on a scale ranging from 0 to 5. Ratings of 0 meant no effect and of 5 complete inhibition. Each treatment was repeated seven times.

Dose-response curves were obtained for seed germination and radicle growth, and the ED_50_ and ED_80_ (concentrations required to obtain 50% and 80% inhibition, respectively) were estimated.

### Post-emergence bioassays: Watering *vs*. spraying

Lettuce seeds were germinated in plastic trays with 5 cm depth layer perlite watered with half-strength Hoagland nutrient solution [29] every other day. Seedlings were grown in a growth chamber at 75% relative humidity and 12 h light at 18°C and 12 h dark at 8°C for three weeks. Plants with three fully expanded leaves were transferred to pots containing perlite watered with half-strength Hoagland nutrient solution to stimulate root system development, and left to acclimate. One week later, plants were exposed to the different post-emergence treatments: control *vs*. eucalyptus aqueous extract. Treatments were applied in two different forms: watering *vs*. spraying, with the aim to determine the differing effects of entry through the root compared to damage by contact.

For the watering treatment, the aqueous extract prepared at 66.7 g L^-1^ in half-strength Hoagland nutrient solution was applied daily until percolation, using plants watered with half-strength Hoagland solution as a negative control. For the spraying treatment, plants were sprayed once a day with crude extract prepared at 66.7 g L^-1^ in distilled water, using plants sprayed with distilled water as negative control. Lettuce plants were sprayed until all the leaves were covered with small droplets, just before the point of runoff. Sprayed plants were as well watered daily with half-strength Hoagland solution during the experiment. Six replicates per treatment and application form were prepared.

#### Chlorophyll *a* fluorescence measurement

The effects of the imposed treatments were sequentially measured on the photosynthetic efficiency of three lettuce plants per treatment chosen at random, by using a Maxi Imaging PAM Chlorophyll Fluorescence System from Walz (Effeltrich, Germany). Immediately before aqueous extract treatment and every 24 h during 5 days, chlorophyll fluorescence parameters were obtained (Table 1). At each measuring time, a dark acclimation period of 5 min was imposed on the plants to allow all reaction centers to open and minimize fluorescence associated with the energization of the thylakoid membrane [30]. After that, whole plants were successively illuminated at an intensity of 0.5 μmol m^-2^ s^-1^ for the measurement of *F*_*o*_, and with a saturating pulse of 2700 μmol m^-2^ s^-1^ intensity for the measurement of *F*_*m*_. Finally, plants were exposed for 5 min to actinic illumination at 110 μmol m^-2^ s^-1^ (measuring the corresponding fluorescence level, *F*) which was interrupted every 20 s with 800 ms saturating pulses of 2700 μmol m^-2^ s^-1^ for the measurement of *F*_*m*_*´*. From these parameters, other variables (Table 1) were calculated by the instrument software using different formula according to Bilger and Björkman [31], Demmig-Adams *et al*. [32], Genty *et al*. [33] and Kramer *et al*. [34]. Fifteen measurements were obtained for each variable at each measuring time, which yielded a kinetic plot for each parameter along time. The average of the area was calculated from the kinetic measurement for the three replicates in every treatment.

**Table 1:** Chlorophyll fluorescence parameters measured with the Maxi Imaging PAM fluorometer.

*F*	Fluorescence yield	
*F*_*o*_	Dark fluorescence yield	
*F*_*o*_*´*	Fluorescence yield of light-adapted leaves	= *F*_*o*_ / (*F*_*v*_ / *F*_*m*_ + *F*_*o*_ / *F*_*m*_*´*)
*F*_*m*_	Maximal fluorescence yield of dark-adapted leaves	
*F*_*m*_*´*	Maximal fluorescence yield of light-adapted leaves	
*F*_*v*_	Variable fluorescence yield of dark-adapted leaves	= *F*_*m*_ − *F*_*o*_
*F*_*v*_*´*	Variable fluorescence yield of light-adapted leaves	= *F*_*m*_*´* − *F*_*o*_*´*
*F*_*v*_*/F*_*m*_	Maximal (photosystem II) PSII efficiency	= (*F*_*m*_ − *F*_*o*_)/ *F*_*m*_
φ_*II*_	Effective (photosystem II) PSII quantum yield	= (*F*_*m*_*´*−F)/ *F*_*m*_*´*
φ_*NPQ*_	Quantum yield of regulated energy dissipation	= 1- φ_*II*_ -1/ (φ_*NPQ*_ +1+qL(*F*_*m*_ / *F*_*o*_ -1))
φ_*NO*_	Quantum yield of non-regulated energy dissipation	= 1/ (φ_*NPQ*_ +1+ *q*_*L*_ (*F*_*m*_ / *F*_*o*_ -1))
*q*_*N*_	Coefficient of non-photochemical quenching	= (*F*_*m*_ − *F*_*m*_*´*)/ (*F*_*m*_ − *F*_*o*_*´*)
*q*_*L*_	Coefficient of photochemical quenching	= (*F*_*m*_*´*-F)/ (*F*_*m*_*´* -*F*_*o*_*´*) · *F*_*o*_*´*/ *F*
ETR	Estimated rate of photosynthetic electron transport	= 0.5· φ_*II*_ ·PAR^a^·Abs.μequivalents m^-2^s^-1^

^*a*^ PAR, Photosynthetic Active Radiation

#### Photosynthetic pigments content

After 5 days of treatment, each plant measured for chlorophyll fluorescence was analyzed for photosynthetic pigments. Pigments were extracted from 100 mg of fresh material per replicate in 1.5 mL methanol and centrifuged at 3500 g for 5 minutes. Then, 0.5 mL of the supernatant were diluted with extra 0.5 mL of methanol, and chlorophyll a (Chl a), chlorophyll b (Chl b) and carotenoid contents were determined spectrophotometrically (WPA Lightwave Diode-Array UV/Vis) after Wellburn [35].

#### Protein content determination

Total proteins were quantified by using the spectrophotometric Bradford assay as described by Pedrol *et al*. [36], in the same 3 lettuce seedlings used for pigment determination. For each replicate, 100 mg of fresh leaves were homogenized in 0.8 mL of 0.05 M Tris buffer containing 0.05 g of the antioxidant polyvinyl polypyrrolidone (PVPP). The homogenates were centrifuged at 4°C and 14000 *g*_n_ for 20 min. Then, 0.1 mL of the supernatant was mixed with 2 mL of Bradford´s reagent and absorbance was measured at 595 nm against a dose-response curve of commercial bovine seroalbumin (BSA).

#### Growth parameters

The remaining three lettuce plants from each treatment were used to determine the fresh and dry weights of aerial and root biomass, and the length of roots. Fresh leaves and fresh roots from each replicate were weighted before and after being dried at 70°C for 72 h. The dry weight/fresh mass ratio was obtained for aerial biomass.

### Chemical analyses of aqueous extract

#### Reagents and standards

Formic and sulphuric acids and methanol were obtained from LiChrosolv^®^ (Darmstadt, Germany). Phenolic acids and flavonoid standards were purchased from Extrasynthèse (Genay, France), and organic acids from Sigma-Aldrich (St. Louis, MO, USA). The water was treated in a Mili-Q water purification system (Millipore, Bedford, MA).

#### Phenolic compounds identification and quantification by high performance liquid chromatography coupled with a diode array detector (HPLC-DAD)

The crude aqueous extract was freeze-dried (model 4.5 Freezone apparatus, Labconco, Kansas City, MO), obtaining a yield rate of 6.46 g of lyophilized per liter. Twenty-five mg of lyophilized were dissolved in 1 mL of methanol and then filtered by 0.45 μm PTFE membrane. Separation of the phenolic compounds was achieved by analyzing 20 μL of the methanol extract on an analytical HPLC unit (Gilson), with a reversed-phase Spherisorb ODS2 column (250 x 4.6 mm, 5 μm particle size; Waters, Milford, MA, USA) with a C18 guard column. The solvent system used was carried out according to a procedure described by Almeida *et al*. [16], using a mixture of water/formic acid (19:1 vol/vol; solvent A) and methanol (solvent B). The gradient was as follows: 0 min, 5% B; 3 min, 15% B; 13 min, 25% B; 25 min, 30% B; 35 min, 35% B; 39 min, 45% B; 42 min, 45% B; 44 min, 50% B; 57 min, 55% B; 60 min, 70% B; 66 min, 75% B; and 70 min, 100% B; 75 min, 100% B; 76 min, 5% B. Elution was performed at a solvent flow rate of 1 mL/min. Detection was achieved with a Gilson diode array detector. Spectral data from all peaks were collected in the range of 200–400 nm and chromatograms were recorded at 320 nm and at 350 nm, as these correspond to highest absorption of the identified phenolic acids and flavonoids, respectively. The data were processed by Unipoint System software (Gilson Medical Electronics, Villiers le Bel, France). The compounds were identified by comparing their elution order and UV-vis spectra with authentic standards. Peak purity was checked by the software contrast facilities. Phenolic compounds quantification was achieved by the absorbance recorded in the chromatograms relative to external standards, using the following equation:

$$C\left(c\right)=\frac{A\left(c\right)}{A\left(st\right)}\times C\left(st\right)$$

Where *C(c)* is the concentration of the compound in the sample, *A(c)* is the peak area of the compound in the sample chromatogram, *C(st)* is the concentration of the standard in the reference solution and *A(st)* is the area of the peak for the standard in the reference chromatogram. This procedure was performed in triplicate. Data presented are the mean values of three independent measurements.

#### Organic acids identification and quantification by HPLC-UV

Twenty five mg of lyophilized aqueous extract were dissolved in 1 mL 0.01 N H_2_SO_4_, followed by filtration by a 0.45 μm Nylon membrane. The separation and quantification of organic acids were carried out according to the procedure described by Oliveira *et al*. [37], in a system consisting of an analytic HPLC unit (Gilson Inc., Middleton, WI) with an ion exclusion Nucleogel Ion 300 OA (300×7.7 mm; Macherey-Nagel, Düren, Germany) column. Elution was performed in isocratic mode with H_2_SO_4_ (0.01 N), at a flow rate of 0.2 mL/min. Detection was achieved with a UV detector set at 214 nm. Organic acids quantification was done by measuring the absorbance recorded in the chromatograms relative to external standards. This procedure was performed in triplicate. Data presented are the mean values of three independent measurements.

### Statistical analyses

Replicated experiments were conducted in a completely randomized design. After testing for normality by Kolmogorov-Smirnov test and for homogeneity of variance by Levene´s test, means were analyzed using independent samples Student´s *t*-test or one-way ANOVA at *P* ≤0.05. LSD test (*P* = 0.05) was used for multiple comparisons of means. In the case of heteroscedasticity, Kruskal-Wallis test and U of Mann-Whitney were used. Semi-quantitative data obtained from *A*. *stolonifera* was subjected to Median test regarding the discontinuity of values, besides U of Mann-Whitney for post hoc comparisons. Dose-response curves were modeled by regression analysis with mathematical models, and the most appropriate model was selected for each case on the basis of the determination coefficient [3]. Statistical analyses were performed using the IBM SPSS Statistics 19 software (IBM SPSS Inc., Chicago, IL, USA).

## Results

### Pre-emergence dose-response bioassays

**Fig 1:**
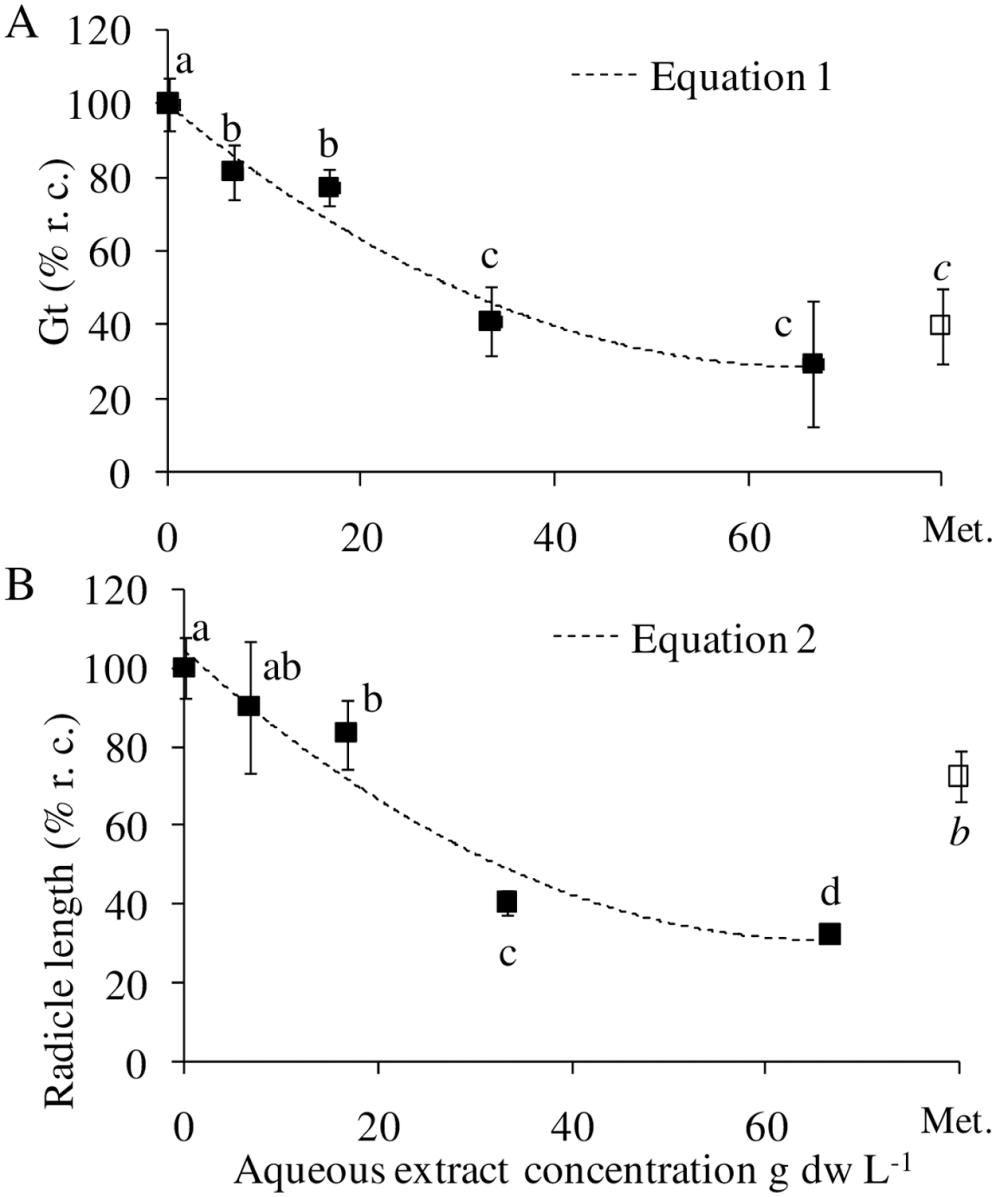
Dose-response curves for *E*. *globulus* aqueous extract on (A) germination and (B) radicle length of *L*. *sativa*. Mean values labeled with distinct letters are significantly different at *P* ≤ 0.05 (LSD post hoc multiple comparisons). Symbols represent mean values of four replicates ± SD. Met., metolachlor (label dose); % r.c., percentage with respect to the control.

The aqueous extract of *E*. *globulus* affected significantly the germination and radicle length of lettuce in a concentration-dependent manner (Fig 1). Using the best-fit equation based on the coefficient of determination (*r*^*2*^), the obtained ED_50_ and ED_80_ values for germination and radicle growth of lettuce are shown in Table 2.

**Table 2:** *E*. *globulus* leaf aqueous extract ED values for germination and radicle length of *L*. *sativa*.

	Regression equation		*r*^2^ ^a^	ED_50_ ^b^ (g dw L^-1^)	ED_80_ ^c^ (g dw L^-1^)
Total germination	*y* = 0.016 *x*^2^–2.161 *x +* 99.93	[1]	0.963	29.6	o.r. ^d^
Radicle length	*y* = 0.016 *x*^2^–2.214 *x* + 104.1	[2]	0.941	31.7	o.r. ^d^

^*a*^ *r*^2^ = determination coefficient

^*b*^ ED_50_, ^*c*^ ED_80_ = concentration required to obtain 50% and 80% germination, radicle or shoot length inhibition, respectively.

^*d*^ o.r. = out of range

Mean Gt and radicle growth values of the different eucalyptus concentrations (Fig 1) ranged from 82 to 30% and 90 to 33% of control, respectively, with a highly significant effect of concentration (ANOVA, *P* ≤ 0.01 for Gt; *P* ≤ 0.001 for growth). Regardless no concentration attained the ED_80_ value (Table 2), eucalyptus concentrations above 20 g dw L^-1^ were significantly more inhibitory of growth than metolachlor: eucalyptus at 33.3 to 66.7 g dw L^-1^ inhibited 60 to 68% of control, whereas the synthetic herbicide at 0.153 g L^-1^ attained 28% inhibition (Fig 1).

**Fig 2:**
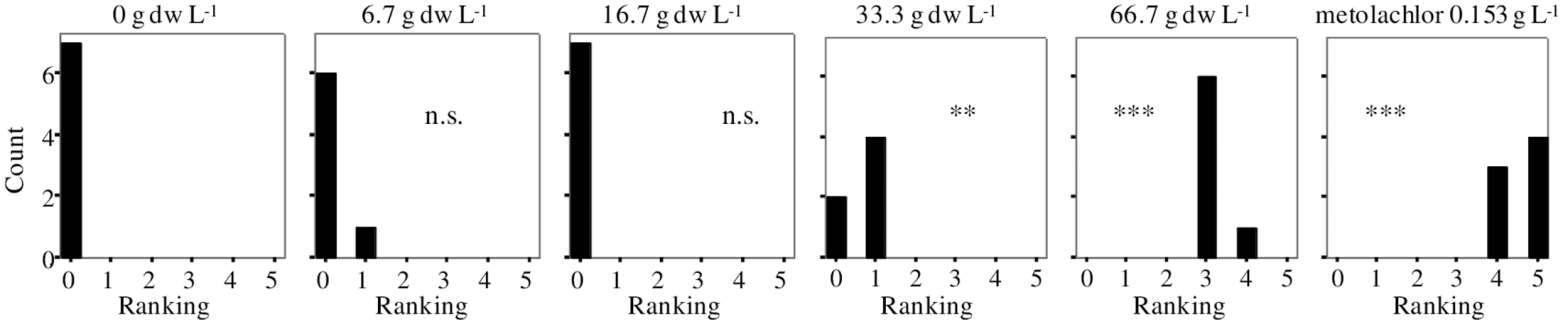
Effects of *E*. *globulus* aqueous extract at different concentrations and metolachlor (label dose) on the growth of *A*. *stolonifera* ranking from 0 to 5: 0, no effect; 5, total inhibition. Count cases, n = 7. Asterisks indicate significant differences between control and each treatment (*P ≤ 0.05, **P ≤ 0.01, ***P ≤ 0.001, n.s. not significant *P* > 0.05; Mann-Whitney U test).

Results of phytotoxicity assessment against the model monocotyledon *A*. *stolonifera* are shown in Fig 2. Analysis of semi-quantitative data showed also highly significant effects of concentration (Median test, *P* ≤ 0.001) on this STS. Statistical comparisons with control revealed very significant growth reductions at 33.3 g dw L^-1^ for eucalyptus extract, and a highly significant inhibition at 66.7 g dw L^-1^ similar to that imposed by metolachlor 0.153 g L^-1^, which did not completely inhibit the growth of bentgrass seedlings as well.

### Post-emergence bioassays: Watering *vs*. spraying

#### Chlorophyll a fluorescence measurement

**Fig 3:**
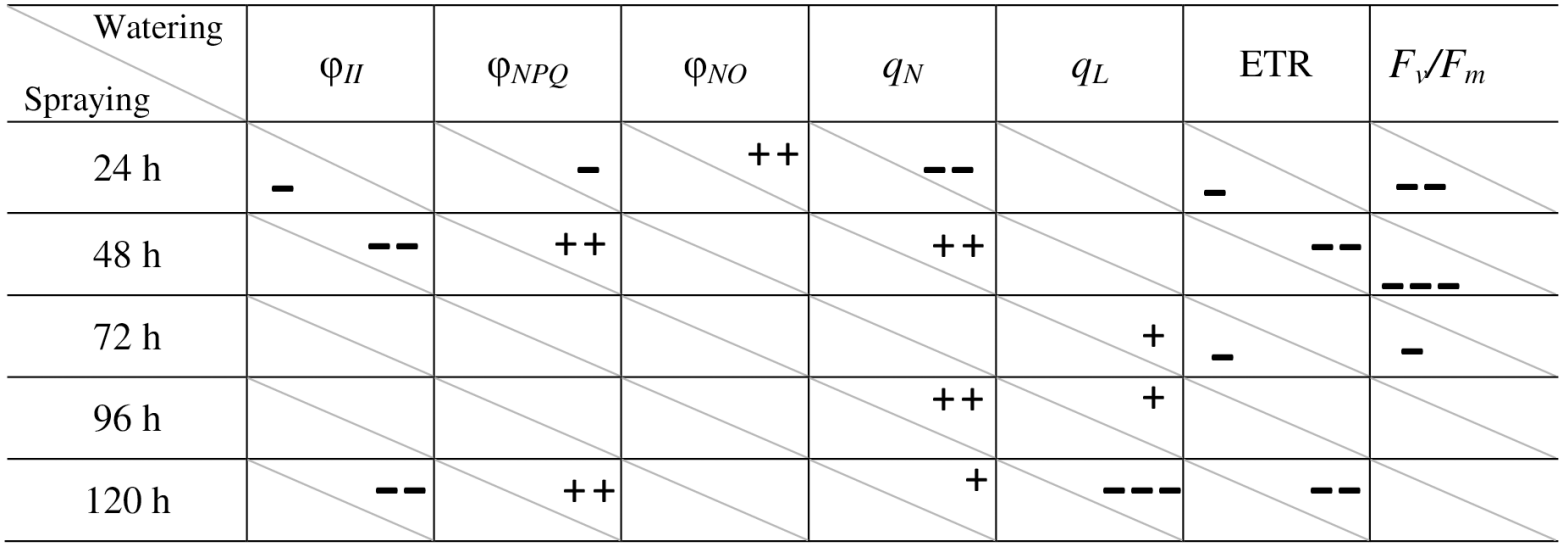
Significative differences of photosynthetic parameters on *L*. *sativa* adult plants treated with *E*. *globulus* aqueous extract by spraying vs. watering. Positive (+) and negative (-) signs indicate increase or decrease with respect to the control. The number of signs indicates the level of statistical significance: one, *P* ≤ 0.05; two, *P* ≤ 0.01; three, *P* ≤ 0.001; independent samples *t*-test.

*E*. *globulus* aqueous extract affected the photochemical and non-photochemical status of the treated plants, with interactions depending on the mode of application (Fig 3). In eucalyptus-watered plants, *in vivo* measurements at the first 24 h after application revealed an initial damage (Fig 3) related to significant increases in fluorescence emission (φ_*NO*_) (*P* ≤ 0.01) to the detriment of heat dissipation (φ_*NPQ*_ and *q*_*N*_). There were significant reductions in photochemical quenching (φ_*II*_) and ETR (*P* ≤ 0.01) at 48 h, with concomitant increases in φ_*NPQ*_ and *q*_*N*_ values. This increase was followed by a stimulation of the photosynthetic activity (*q*_*L*_) after 72 h and 96 h of treatment, which dropped significantly at the end of the assay (φ_*II*,_ *q*_*L*_ and ETR), whereas φ_*NPQ*_ and *q*_*N*_ increased again. The maximum PSII efficiency (*F*_*v*_*/F*_*m*_) was not affected. Nonetheless, in sprayed plants, the photosynthetic activity (φ_*II*,_ ETR and *F*_*v*_*/F*_*m*_) fell during the first 72 h, and then *F*_*v*_*/F*_*m*_ recovered.

#### Growth parameters, photosynthetic pigments, and protein contents

**Fig 4:**
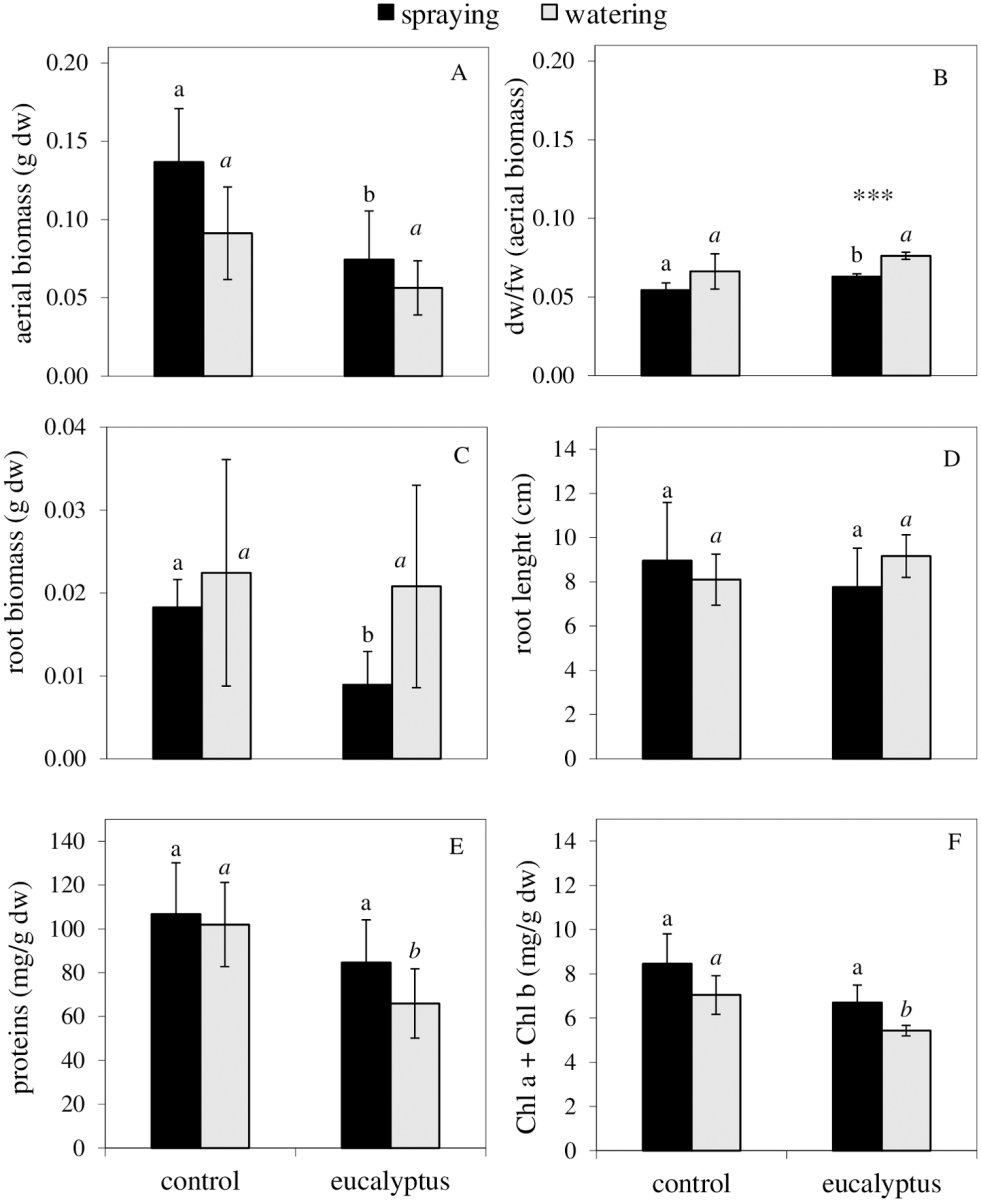
Phytotoxic effects of eucalyptus aqueous extracts on growth and physiological parameters of lettuce seedlings. Bars represent mean values of three replicates ± SD. For each mode of application, mean values labeled with distinct letters are significantly different (*P* ≤ 0.05; independent samples t-test). For each treatment, asterisks indicate significant differences between watering and spraying (*P ≤ 0.05;**P ≤ 0.01;***P ≤ 0.001; independent samples t-test).

Parameters measured on lettuce plants after harvesting are summarized in Fig 4. Eucalyptus-treated plants suffered conspicuous effects on morphological and biochemical variables measured at 5 days of treatment. When applied by spraying, eucalyptus aqueous extract had a significant effect on growth, reducing the aerial and root biomass (Fig 4A and 4C) and increasing the dry weight/fresh weight ratio (Fig 4B). When applied by watering, eucalyptus plants had similar or higher mean values of root biomass (Fig 4C) and length (Fig 4D) than those of control, whereas differences did not achieve statistical signification. However, the application by watering produced significant effects on the measured metabolites, thus reducing protein contents (Fig 4E) and chlorophyll concentrations (Fig 4F) with respect to control. Statistical analysis also revealed significant differences between application methods for dw/fw ratio of aerial biomass (*P* ≤ 0.001) in lettuce plants treated with eucalyptus aqueous extract (Fig 4B). Finally, no significant effects on carotenoid levels were observed between treatments or modes of application and neither visual symptoms of damage as leaf chlorosis or necrosis.

### Chemical composition of eucalyptus aqueous extracts

From the HPLC analyses, 8 phenolic compounds and 5 organic acids were identified (Table 3). The phenolic profile of eucalyptus extract revealed the presence of three hydroxycinnamic acids (chlorogenic and two ρ-coumaric derivatives), and five flavonoids (ellagic acid, hyperoside, rutin, quercitrin, and kaempferol 3-O-glucoside). In relation to organic acids, shikimic and succinic acids were the major compounds (55.4%). For these two compounds, as well as for ellagic acid and kaempferol 3-O-glucoside, the HPLC-DAD did not throw a well-defined separation, making them not quantifiable as single compounds.

**Table 3:** Chemical profile of *Eucalyptus globulus* aqueous extract.

Compound	RT ^a^	Content (μg ·g^-1^ of lyophilized extract) ^b^	Content (μg ·mL^-1^ of extract) ^c^	% of each compound respect to the total
*Phenolic compounds*				
Chlorogenic acid	15.6	1808.6 ± 1.6	11.7 ± 0.01	15.3
ρ-coumaric acid derivative ‘1’	17.5	242.3 ± 1.7	1.6 ± 0.01	2.1
ρ-coumaric acid derivative ‘2’	19.9	174.2 ± 0.2	1.1 ± 0.001	1.5
Hyperoside	38.1	2997.9 ± 9.4	19.4 ± 0.06	25.4
Rutin	39.6	1713.5 ± 10.8	11.1 ± 0.07	14.5
Quercitrin	43.2	648.2 ± 28.4	4.2 ± 0.18	5.5
Ellagic acid + Kaempferol 3-O-glucoside	43.7	not quantifiable		61.6
*Organic acids*				
Citric acid	30	22493.2 ± 1745.6	145.3 ± 11.3	24.4
Malic acid	36.1	13891.9 ± 630.6	89.7 ± 4.1	15.1
Shikimic + Succinic acid	47.3	not quantifiable		55.4
Fumaric acid	62.1	4676 ± 164.5	30.2 ± 1.1	5.1

^*a*^ RT = retention time in min

^*b*^ Content (μg ·g^-1^ of lyophilized extract) = quantity of each compound present in 1 gram of lyophilized extract

^*c*^ Content (μg ·mL^-1^ of extract) = quantity of each compound present in 1 mL of extract according with the yield of the lyophilized

## Discussion

Germination and growth bioassays on lettuce and bentgrass as model target species, as well as tests measuring biochemical parameters and photosynthetic activity, have been frequently used to assess phytotoxicity of plant extracts [38–40]. In the same scenario, the measurement of physicochemical properties of aqueous extracts is commonly explored, since they can cause changes in cellular processes that could be misled as a possible phytotoxic effect. Taking into account that pH values between 5 and 7 [25], EC values below 2 dS m^−1^ [41] or osmolarity values of PEG lower than 0.1 osmol kg^-1^ [42] do not affect lettuce development, any inhibitory effect observed in our pre and post-emergence bioassays could be attributed *a priori* to the phytotoxins present in the plant aqueous extract. In our pre-emergence bioassays, the observed phytotoxicity confirmed the previous reports by Souto *et al*. [22] and Yamagushi *et al*. [10] wherein *E*. *globulus* aqueous extract inhibited lettuce germination and radicle growth. A progressive increase in inhibition of germination and radicle length was observed concomitantly to increase in extract concentration. For these cases, dose-response curves are useful for estimating parameters ED_50_ and ED_80_, which are important because they establish a frame of reference to which subsequent tests can be compared [28]. Furthermore, the identification of the phytotoxic dose of an aqueous extract could be useful for further bioassays in greenhouse and field conditions.

To determine the effect of post-emergence-applied aqueous extract, “the measurement of chlorophyll *a* fluorescence emitted by intact, attached leaves is thought to be a reliable, non-invasive method for monitoring photosynthesis and for judging the physiological status of the plant” [43]. For the assessment of post-emergence bioherbicide potential, the test performed with lettuce adult plants was useful to study the possible sites of action of the plant extracts, considering that their effects and mechanisms of action can be completely different in adult plants than in seedling metabolism, as well as depending on the application method [44]. Chlorophyll contents are closely related to the absorption and emission of energy, and any change in them is expected to lead a change in photosynthesis [45]. As stated Zhou and Yu [45], photoinhibition of photosynthesis by allelochemicals is typically characterized as a reduction in quantum yield of PSII photochemistry and a decrease in Chl fluorescence. Reduction in quantum yield can be evaluated by the ratio of variable to maximum fluorescence (*F*_*v*_*/F*_*m*_). If a plant cannot emit the excess energy received from the sun, this excess energy is transferred to oxygen via chlorophyll, resulting in photo-oxidation damage. Excessive damage leads to membranes destruction and chlorophyll oxidation. In our bioassay, watering application of eucalyptus aqueous extract revealed more general conspicuous phytotoxic effects than the spraying method. A reduction in photochemical quenching (φ_*II*_) and ETR, with the decline of chlorophyll *a* + *b* was detected in watered-plants, which indicates that the biochemical phase of photosynthesis was directly affected by the exogenous application of eucalyptus; however, lettuce plants could activate mechanisms of regulated energy dissipation in the form of heat (increase in φ_*NPQ*_ and *q*_*N*_) and then acclimate [43]. In agreement with our results, Batish *et al*. [46] found that total chlorophyll content and respiratory activity were reduced in response to eucalypt oil in all tested species. Similarly, extracts of abscised leaves of eucalyptus have been shown to reduce chlorophyll content on crops, effect that was related to a possible poor photosynthesis and, as a result, a poor plant growth [21]. Together with the effects described on photosynthesis and chlorophyll contents, protein concentration was also significantly reduced in our eucalyptus-watered plants. Those effects might indicate an inhibition of the synthesis of these compounds in the leaves as a symptom of early stress caused by phytotoxicity, which is still not reflected at whole plant growth level. These results are in conformity with those of Djanaguiraman *et al*. [47], who detected reductions in chlorophyll and protein content in blackgram, rice and sorghum seedlings by *E*. *globulus* leachates assayed at different concentrations. In our case of spraying application, and differently to watering, eucalyptus aqueous extract significantly inhibited plant growth, and increased dry weight/fresh weight ratio of aerial biomass, without affecting protein or chlorophyll contents. Such decrease in growth can be a consequence of the early inhibition of photosynthetic efficiency measured as *Fv/Fm*, which was then restored as a symptom of acclimation. Root biomass resulted as well diminished in sprayed-plants, suggesting a systemic behavior. Such observations could be reflecting different modes of action depending on the site of phytotoxins entry, growth and morphological parameters being early affected by spraying on leaves, and physiological traits more affected by watering.

Despite the conspicuous inhibition or reduction detected for some physiological and/or morphological parameters, external symptoms of damage as chlorosis or necrosis were absent in lettuce adult plants. So, the lack of remarkable effects on lettuce, being a standard target species chosen for its high sensitivity to allelochemicals, led us to discard the use of eucalyptus aqueous extract as a post-emergence bioherbicide, which effects would be agronomically irrelevant in the context of weed control.

In the present study, we have used natural aqueous extracts because water is the extraction solvent in nature, so the use of aqueous extraction simulated the natural release of water-soluble phytotoxins into the environment. Moreover, the advantage in utilizing aqueous extracts is given by the joint action of several compounds present in them and not only by a simple compound, considering that many authors demonstrated that a mixture of compounds could be more phytotoxic than their respective individual compounds [3,48,49]. Most of the germination and growth inhibitors present in aqueous extracts are phenolic compounds [50], and have been widely reported to affect many physiological processes [45,51], *viz*. phytohormone activity, mineral uptake, plant water balance and stomatal function, photosynthesis, respiration, organic synthesis of certain compounds and flow of carbon [52]. *Via* HPLC analysis, the major phytotoxic substances identified in our eucalyptus leaf aqueous extract were hyperoside and the mixtures of ellagic acid-kaempferol 3-O-glucoside and shikimic-succinic acids. Of these, hyperoside was also found as the main flavonoid in the *E*. *globulus* phenolic profile by Deszi *et al*. [18], and the presence of ellagic acid is in conformity with Almeida *et al*. [16], Amakura *et al*. [17], Boulekbache-Makhlouf *et al*. [6] or Souto *et al*. [22]. Most of the compounds identified here are well-known phytotoxins, so their occurrence in our aqueous extract can explain the phytotoxic effects described when applied both in pre- and post-emergence on the STS. Moreover, the release of these and other compounds can underlie the herbicidal activity observed when *E*. *globulus* leaves are incorporated into the soil as a green manure [15]. Chon *et al*. [49] detected chlorogenic and ρ-coumaric acids in *Xanthium occidentale* water fraction and correlated their presence and amount with the phytotoxicity observed from the extract. Mersie and Singh [53] also found an inhibition of photosynthesis in *Abutilon theophrasti* leaf cells by the action of these phenolic acids. Rutin was suggested as the major allelochemical in buckwheat by Golisz *et al*. [54], while Fujii *et al*. [55] observed a significant inhibitory activity when tested against lettuce seedlings. In another report, the effect of ellagic acid on Arabidopsis fresh weight appeared to be mainly due to root damage [56].

Regarding other low molecular weight organic acids, from our knowledge, this is the first time that the organic acid composition from *E*. *globulus* leaf aqueous extract is reported. In other frameworks, Silva *et al*. [57] showed the presence of succinic, fumaric, malic and citric acids in root tips of six eucalyptus species, and suggested that their synthesis and root exudation could contribute to the adaptation of these species to acid Al-toxic soils. Zhang *et al*. [58] showed that both malic and citric acid inhibited SOD activity and reduced the fresh weight of melon, the latter being also an inhibitor of radicle and hypocotyl growth. On the other hand, An *et al*. [48] concluded that succinic acid showed weak phytotoxic strength, however, it was considered as one of the quantity-dominant compounds which makes it as a high contributor to phytotoxicity. Besides the occurrence of these organic acids probably contributing to phytotoxicity, the presence of high amounts of shikimic acid in the eucalyptus extract could be an indication of a high potential for secondary metabolites production, since it is a key intermediate in the formation of numerous types of secondary compounds (including phenolic acids and flavonoids) [59,60].

## Conclusions

The combination of methods carried out in this study has demonstrated that aqueous extract from *E*. *globulus* leaves exhibit phytotoxic effects on germination, growth and some physiological parameters of standard target species. Despite some phytotoxic effects were found on lettuce adult plant physiology and morphology, the use of eucalyptus aqueous extract will be discarded in post-emergence but can be promising as a pre-emergence bioherbicide. It is concluded that *E*. *globulus* synthesizes a cocktail of phytotoxic compounds that can be released from leaf residues applied to the soil, and then interfere with the germination and development of other species.
